# Psychological and physical correlates of musculoskeletal symptoms in male professional divers and offshore workers

**DOI:** 10.1186/2046-7648-2-5

**Published:** 2013-02-01

**Authors:** John AS Ross, Jennifer I Macdiarmid, Claire L Rostron, Stephen J Watt, John R Crawford

**Affiliations:** 1 Environmental & Occupational Medicine, Section of Population Health, University of Aberdeen School of Medicine and Dentistry, Polwarth Building, Foresterhill, Aberdeen, AB25 2ZD, UK; 2 Obesity and Metabolic Health, Rowett Institute of Nutrition and Health, University of Aberdeen School of Medicine and Dentistry, Aberdeen, AB25 2ZD, UK; 3 Department of Life, Health and Chemical Sciences, Open University, Milton Keynes, MK7 6AA, UK; 4 School of Psychology, University of Aberdeen, Aberdeen, AB25 2UB, UK

**Keywords:** Diving, Symptoms, Musculoskeletal system, Neuropsychological tests

## Abstract

**Background:**

Underwater divers are more likely to complain of musculoskeletal symptoms than a control population. Accordingly, we conducted a study to determine whether musculoskeletal symptoms reflected observable physical disorder, to ascertain the relationship between symptoms and measures of mood, memory and executive function and to assess any need for future screening.

**Methods:**

A 10% random sample of responders to a prior postal health questionnaire was examined (151 divers, 120 non-diving offshore workers). Participants underwent physical examination and a neuropsychological test battery for memory and executive function. Participants also completed the Hospital Anxiety and Depression Scale for anxiety (HADSa) and depression (HADSd), and questionnaires for physical health-related quality of life (SF36 PCS), mental health-related quality of life (SF36 MCS), memory (Cognitive Failures Questionnaire (CFQ), Prospective and Retrospective Memory Questionnaire (PRMQ)), executive function (dysexecutive syndrome questionnaire (DEX)), musculoskeletal symptoms (MSS) and general unrelated symptom reporting.

**Results:**

Of participants with moderate/severe musculoskeletal symptoms, 52% had physical signs, and of participants with no symptoms, 73% had no physical signs. There was no difference in the prevalence of signs or symptoms between groups. Musculoskeletal symptoms were associated with lower SF36 PCS for both groups. In divers, musculoskeletal symptoms were associated with higher general unrelated symptom reporting and poorer scoring for HADSa, PRMQ, CFQ and DEX with scores remaining within the normative range. A positive physical examination was associated with general unrelated symptom reporting in divers. There were no differences in neuropsychological test scores attributable to either group or musculoskeletal symptoms.

**Conclusions:**

Musculoskeletal symptoms were associated with physical signs, but this was not a strong effect. Reporting of musculoskeletal symptoms by the divers studied was also associated with a tendency to report symptoms generally or somatisation, and caution should be exercised regarding their interpretation as an indication of physical disease or their use for health screening.

## Background

There are several reports of occupation-related musculoskeletal disorder in professional divers. Dysbaric bone necrosis is a recognised industrial disease for divers with a risk of shoulder and hip arthritis. In a UK workforce, however, clinical problems arising from this condition are uncommon [1]. Independently of bone necrosis, Hoiberg showed decompression illness to be a risk factor for pain in the joints and limbs [2] and found higher hospitalisation rates for joint disorders in military divers [3,4]. Another study found that 60% of Norwegian commercial divers working in the North Sea oil industry from 1965 to 1990 reported frequent joint pains [5]. Finally, a questionnaire-based cross-sectional study of UK professional divers’ health status in relation to a non-diving control group of offshore oil industry workers found that divers reported musculoskeletal symptoms more frequently than controls [6]. Currently, the offshore oil and gas industry is developing health surveillance techniques for its diving workforce since they are a legal requirement for all workers in Norway and physical factors in the diving work environment, such as noise and vibration exposure, might justify such measures more generally. The studies referenced could indicate that screening for musculoskeletal problems may be a reasonable part of this exercise. Musculoskeletal symptom reporting, however, can also be associated with disorders of mood [7-9], and there are indications of a somatising tendency in offshore oil industry divers which could underlie increased symptom reporting [6]. Divers are also more likely to have cognitive symptoms of ‘loss of concentration or forgetfulness’ [6]. Accordingly, we conducted a study that aimed to determine the relationship of musculoskeletal symptom reporting with observable physical disorder, general symptom reporting, mood and subjective and objective measures of memory and executive function in divers and offshore workers.

## Methods

Participants were age-matched male professional divers (*n* = 151) and non-divers (*n* = 120) who had previously completed a health questionnaire study [6]. Divers had a professional diving certificate registered with the UK Health and Safety Executive before 1991. Non-divers were oil and gas industry offshore workers who had undergone a fitness-to-work medical examination between 1990 and 1992 and had never dived. To reduce any effect of survivor bias, participants included men both currently working in the diving or offshore industry and who had retired from these industries. Participants were randomly selected from samples of 1,035 offshore workers (517 contacted) and 1,540 professional divers (386 contacted) until an approximate 10% sample was established. The two groups were similar in terms of lifestyle (Table 1) with most participants in employment. Non-divers retired from their industry, while divers were more likely to move on to another job. In the diver group, 93 participants (62%) had worked in the offshore oil industry, but many divers had worked in several different sectors (Table 2). Two characteristics were of importance in this study of musculoskeletal symptoms. There was no difference in the reported prevalence of arthritis between the groups studied. Subjects in the diver group reported a statistically significant lower prevalence of receiving medication or medical treatment. 

**Table 1:** Characteristics of divers and offshore workers

	**Divers(*n*= 151)**	**Offshore workers(*n*= 120)**	***p*value**
Age (mean (95% CI))	46.9 (45.7–48.2)	46.3 (44.9–47.7)	0.866
Cigarette pack years (median (IQR))	0.6 (0.0–12.5)	0.0 (0.0–20.0)	0.759
Units of alcohol per year (median (IQR))	612 (240–981)	480 (210–956)	0.455
BMI (kg/m^2^) (mean (95% CI))	27.6 (27.0–28.2)	27.6 (26.7–28.4)	0.487
Years as a diver or offshore worker	17.3 (16.0–18.6)	16.5 (15.3–17.7)	0.403
(mean (95% CI))			
Medication or medical treatment (% (*n*))	20 (29)	31 (37)	0.033
Diagnosed arthritis (% (*n*))	8 (12)	6 (7)	0.530
Employment status (% (*n*))			
Still working as a diver or offshore worker	47 (74)	80 (89)	<0.001
Other employment	35 (53)	6 (7)	0.236
Employed	89 (133)	87 (104)	
Unemployed	2 (3)	3 (4)	
Not working - on sickness benefit	0 (0)	3 (3)	
Retired	9 (14)	8 (9)	
Retirement status (% (*n*))			
Not due to illness	6 (9)	4 (5)	0.575
Due to ill health - caused by diving	1 (2)	-	
Due to ill health - unrelated to diving	2 (3)	3 (4)	

95% CI, 95% confidence interval; IQR, interquartile range; BMI, body mass index; *p*, probability.

**Table 2:** Industrial sector in which divers had worked

**Industrial sector**	**Number of divers**
Offshore oil and gas	93 (62%)
Coastal and inshore	117 (78%)
Shellfish harvesting	28 (19%)
Scientific	26 (17%)
Police	43 (29%)
Media	24 (16%)
Recreational instructor	32 (22%)
Military	35 (23%)
Other	12 (8%)

Smoking was assessed by calculation of pack years. Alcohol habit was assessed by the number of units consumed over a 1-year period allowing for time spent in an alcohol-free working environment. Each participant completed the Short Form 36 health-related quality of life questionnaire from which summary scores for physical quality of life (SF36 PCS) and mental quality of life (SF36 MCS) were derived [10]. The Hospital Anxiety and Depression Scale (HADS), the Cognitive Failure Questionnaire (CFQ), the Prospective and Retrospective Memory Questionnaire (PRMQ) and a dysexecutive syndrome questionnaire (DEX) were also completed. The HADS was designed to identify cases of anxiety disorder (HADSa) and depression (HADSd) in non-psychiatric hospital and clinic patients [11] and has been validated for comorbidity screening in musculoskeletal disease [12,13]. The CFQ assesses self-reported failures of perception and motor behaviour in addition to memory failures. It has been shown to correlate more highly with executive functions rather than tasks of memory and is argued to measure failure in the control of attention and memory [14]. The PRMQ assesses everyday memory failures, and factor analysis of the PRMQ has confirmed that the questionnaire assesses a single common factor of memory [15]. The DEX forms part of the Behavioural Assessment of the Dysexecutive Syndrome test battery [16]. The questionnaire asks about four broad areas of executive dysfunction: emotional or personality changes, motivational changes, behavioural changes and cognitive changes, and generates a single summary score.

Neuropsychological testing methods have been described elsewhere [17] and are summarised here. The Logical Memory (LM) test, from the Wechsler Memory Scale III, and the California Verbal Learning Task (CVLT) II were used to measure immediate and delayed memory. The Cambridge Neuropsychological Test Automated Battery (CANTAB) (CeNeS, Cambridge, UK), a computerised test battery, assessed performance of memory, attention and executive function. The CANTAB tests included the five-choice reaction time, rapid visual process task, spatial recognition memory, stockings of Cambridge and spatial working memory. Current IQ measured using the Wechsler Abbreviated Scale of Intelligence (WASI) gave an estimate of global intellectual function (full score) and two sub-components of current intelligence: Matrix Reasoning (fluid intelligence) and Vocabulary (crystallized intelligence). The data gathered from this test battery were standardised (*z* scores) and used to generate summary/composite scores reflecting memory performance (MemComp) and executive function (ExecComp) as described elsewhere [17]. Neuropsychological tests were always presented in the same order (CANTAB, CVLT, LM, WASI) using standardised instructions. Test assessors were blind to the subject group. Subjects were asked to refrain from drinking alcohol for 24 h prior to the study.

Physical examination was carried out by one of six doctors who were specialist registrars in general medicine or general practitioners with no background in occupational or diving medicine and without other involvement in the research. A *pro forma* physical examination was carried out using standard methods [18] after a period of instruction in the methods to be used. The locomotor system was examined for abnormality of gait and stance, deformity, range of movement and pain or tenderness of the joints of the limbs and the cervical, thoracic and lumbar spine. The examination was noted as normal or abnormal, and abnormality of the spine, hands, upper limbs (excluding the hands) and lower limbs and any pain or tenderness were recorded. A structured medical history was then taken for joint, back pain or neck pain and muscle stiffness at four levels: not at all, slight, moderate, severe (scoring 0–3), and for general symptoms. Reporting of other general (non-musculoskeletal) symptoms was used to give a general symptom score (GSS) of 0–21 (Table 3) in which internal consistency was high enough to allow its use to compare groups (Cronbach’s alpha 0.72). Similar scoring systems have been used elsewhere to assess somatisation [19,20] or the tendency to experience psychosocial stress in the form of physical symptoms. 

**Table 3:** General symptom scoring

**Symptom**		**Score**
General	Breathlessness, cough, wheeze, forgetfulness, loss of concentration, rash, itch or impaired hearing	0–1 for presence or absence of each symptom
Cardiovascular	Ankle swelling, palpitations, orthostatic dyspnoea, nocturnal dyspnoea, chest pain on exertion, claudication	0–1 for presence or absence of any symptom
Respiratory	Sputum production, pleuritic chest pain, haemoptysis	0–1 for presence or absence of any symptom
Alimentary	Dysphagia, indigestion, heartburn, abdominal pain, weight loss, change in bowel habit	0–1 for presence or absence of any symptom
Urogenital	Dysuria, frequency, prostatic symptoms, impairment of sexual function	0–1 for presence or absence of any symptom
Central nervous system	Headaches, fits, faints, tingling, numbness, muscle weakness, excessive thirst, abnormal sleep patterns	0–1 for presence or absence of any symptom
Endocrine	Heat intolerance, cold intolerance, change in sweating, prominence of the eyes, swelling in the neck	0–1 for presence or absence of any symptom
Visual	Eye pain, disturbance of vision	0–1 for presence or absence of any symptom
Hearing	Difficulty hearing, requiring radio or television sound turned up, difficulty detecting direction of sound, dizziness or vertigo, noises in the ears, pain in the ears	0–1 for presence or absence of each symptom

Maximum score = 21.

Neck pain, back pain, joint pain, and muscle stiffness scores were compared and then aggregated to give a four-point musculoskeletal symptom score representing: no symptom, any mild symptom, any moderate symptom or any severe symptom. In order to simplify assessment of factors associated with musculoskeletal symptom reporting, these categories were further collapsed to no symptoms or mild symptoms and moderate or severe symptoms.

In general, discrete variables were assessed using chi-squared tests or binomial logistic regression, and continuous variables were assessed using Student’s *t* tests. The Spearman or Pearson correlation coefficients were used to assess univariate relationships between variables as appropriate. SPSS version 14.0 (SPSS Inc., Chicago, IL, USA) was used for analyses. The general level for reporting statistical significance was taken as *p* ≤ 0.05. For multiple *t* test comparisons, the level for reporting statistical significance was taken as *p* ≤ 0.005 using a Bonferroni correction for multiple comparisons.

The study successfully underwent ethics committee appraisal and received a favourable opinion from the Grampian Region Joint Ethics Committee. This research work was funded by the UK Health and Safety Executive.

## Results

There were no differences between groups in the findings on physical examination. Abnormalities were found in 45 offshore workers (38%) and 60 divers (40%) with pain or tenderness being found in 20 offshore workers (17%) and 33 divers (22%). Overall findings were abnormality of the spine (21%), hands (6%), upper limbs (14%) or lower limbs (20%) with no differences in these rates between groups.

There was no significant difference in musculoskeletal symptom scores between the groups. For moderate to severe symptoms in divers, 6% had neck pain, 13% had back pain, 19% had joint pain, 6% had muscle stiffness and 32% had any moderate or severe symptoms. For offshore workers, 6% had neck pain, 13% had back pain, 20% had joint pain, 5% had muscle stiffness and 36% had symptoms to a moderate or severe degree.

Musculoskeletal symptom score correlated with the prevalence of musculoskeletal abnormality on examination (Spearman coefficient 0.33, *p* < 0.001). Of participants with moderate or severe symptoms (across both occupational groups), 52% had an abnormality on examination. Of participants without such symptoms, 73% had no abnormality. There was no difference in this between the groups. Moderate or severe symptoms of joint pain (*p* < 0.001), muscle stiffness (*p* = 0.01) and neck pain (*p* = 0.04) were associated with abnormalities on examination, but back pain symptoms were not (*p* = 0.50). Moderate or severe symptoms were not related to job status for offshore workers, but divers who had retired permanently from diving were more likely to have symptoms than active divers (*n* = 32, mean odds ratio (OR) 2.15, 95% CI 1.02 to 4.54, *p* = 0.04). Divers with experience of police diving (*n* = 43, mean OR 3.00, 95% CI 1.11 to 7.92, *p* = 0.03) or of diving in the offshore oil and gas industry (*n* = 57, mean OR 3.48, 95% CI 1.20 to 10.15, *p* = 0.02) were more likely to express moderate to severe symptoms.

The relationships between the variables measured and musculoskeletal symptoms and signs for divers and offshore workers are summarised in Tables 4 and 5. Moderate or severe symptoms were associated with reduced SF36 PCS for both divers and offshore workers (Table 4). For divers, however, symptoms were also associated with unfavourable changes in HADSa, CFQ, PRMQ, DEX and increased general symptom reporting. Again, a positive physical examination was only associated with a reduced SF36 PCS for offshore workers (Table 5), but the only significant association for divers, after Bonferroni correction, was with GSS. In divers, this association between general symptom reporting and a positive physical examination was significant when pain and tenderness on examination was present (*n* = 33, *t* = −3.58, *p* = 0.001) but not when it was absent (*n* = 27, *t* = −0.12, *p* = 0.88). These associations were not present in offshore workers. There were no significant differences associated with moderate to severe musculoskeletal symptoms or a positive physical examination and scoring from any the individual neuropsychology tests used in either divers or offshore workers, and this is reflected in the composite scores (Tables 4 and 5).

**Table 4:** Summary of questionnaire and neuropsychological test data in relation to expression of musculoskeletal symptoms

	**Offshore workers**			**Divers**		
	**No symptoms*n*= 82**	**Symptoms*n*= 38**	***p***	**No symptoms*n*= 103**	**Symptoms*n*= 48**	***p***
GSS	3.0 (2.3 to 3.7)	3.9 (2.4 to 5.3)	0.22	2.5 (2.0 to 3.2)	6.3 (4.6 to 8.1)	<0.001
SF36 PCS	50.9 (49.1 to 52.7)	43.8 (40.2 to 47.5)	<0.001	52.4 (50.9 to 53.9)	42.9 (39.3 to 46.4)	<0.001
SF36 MCS	52.3 (50.4 to 54.2)	52.4 (50.2 to 54.8)	0.93	52.3 (51.9 to 54.7)	47.6 (43.7 to 51.5)	0.001
HADSa	5.76 (4.94 to 6.58)	6.58 (5.57 to 7.59)	0.24	6.2 (5.6 to 6.9)	8.0 (6.8 to 9.3)	0.005
HADSd	3.45 (2.78 to 4.12)	4.66 (3.81 to 5.51)	0.04	3.7 (3.1 to 4.2)	5.0 (3.9 to 6.0)	0.02
PRMQ	36.8 (35.0 to 38.6)	40.5 (37.8 to 43.3)	0.02	40.7 (39.1 to 42.3)	47.1 (44.1 to 50.1)	<0.001
CFQ	32.5 (30.0 to 34.9)	36.0 (32.4 to 39.6)	0.11	37.8 (35.3 to 40.2)	44.3 (40.2 to 48.4)	0.005
DEX	17.1 (15.2 to 18.9)	19.0 (16.5 to 21.5)	0.23	18.9 (17.2 to 20.6)	25.6 (21.6 to 29.6)	<0.001
MemComp	0.5 (−0.4 to 1.4)	−0.2 (−1.4 to 1.0)	0.39	0.7 (−0.4 to 1.4)	0.2 (−0.8 to 1.2)	0.46
ExecComp	−0.04 (−0.4 to 0.3)	0.05 (−0.5 to 0.6)	0.78	−0.04 (−0.4 to 0.3)	0.3 (−0.2 to 0.8)	0.25

Data are quoted as mean and 95% CI.

**Table 5:** Summary of questionnaire and neuropsychological test data in relation to positive findings on physical examination

	**Offshore workers**			**Divers**		
	**No signs*n*= 75**	**Signs*n*= 45**	***p***	**No signs*n*= 91**	**Signs*n*= 60**	***p***
GSS	2.5 (1.9 to 3.1)	3.0 (2.1 to 3.9)	0.36	3.7 (3.0 to 4.3)	5.6 (4.5 to 6.8)	0.002
SF36 PCS	51.2 (49.6 to 52.9)	44.4 (40.9 to 47.9)	<0.001	51.1 (49.2 to 53.0)	46.7 (43.7 to 49.8)	0.011
SF36 MCS	52.0 (50.1 to 55.3)	52.9 (50.7 to 55.3)	0.51	52.1 (50.4 to 53.9)	50.4 (47.4 to 53.6)	0.32
HADSa	5.83 (5.07 to 6.58)	6.33 (5.14 to 7.52)	0.45	6.54 (5.71 to 7.19)	7.33 (6.32 to 8.34)	0.15
HADSd	3.73 (3.06 to 4.41)	4.00 (3.09 to 4.91)	0.64	3.91 (3.31 to 4.51)	4.33 (3.44 to 5.23)	0.42
PRMQ	37.6 (35.8 to 39.5)	38.5 (35.8 to 41.2)	0.60	41.4 (39.7 to 43.1)	44.7 (42.0 to 47.5)	0.034
CFQ	33.4 (30.8 to 36.0)	34.0 (30.6 to 37.3)	0.79	39.1 (36.5 to 41.6)	40.8 (37.0 to 44.7)	0.43
DEX	17.7 (15.7 to 19.7)	17.7 (15.3 to 20.0)	0.99	19.6 (17.7 to 21.5)	23.2 (19.8 to 26.5)	0.052
MemComp	0.11 (−0.74 to 0.96)	0.46 (−0.88 to 1.79)	0.65	0.95 (0.18 to 1.73)	−0.09 (−0.95 to 0.78)	0.08
ExecComp	−0.12 (−0.55 to 0.32)	0.16 (−0.31 to 0.62)	0.41	0.07 (−0.25 to 0.40)	0.06 (−0.42 to 0.54)	0.96

Data are quoted as mean and 95% CI.

Within the GSS, reporting moderate to severe musculoskeletal symptoms was significantly associated with respiratory symptoms (*p* = 0.016), urogenital symptoms (*p* < 0.001), central nervous system symptoms (*p* < 0.001), visual symptoms (*p* = 0.008) and endocrine symptoms (*p* = 0.044).

## Discussion

In general, musculoskeletal symptoms in divers and offshore workers had an association with observable physical abnormalities, but the relationship was not strong. The parent questionnaire study [6] found that musculoskeletal symptoms were 7% commoner in divers, and the observation that symptoms do reflect observable abnormalities to some degree might support the concept that musculoskeletal problems may be commoner in divers in a larger population than studied here. Musculoskeletal symptoms, however, were also related to the expression of other unrelated symptoms in divers. Multiple symptom reporting in the absence of identifiable disease can imply a tendency to somatise in a population [20,21], in which case symptom reporting might be taken as an unreliable indicator of physical illness and would not be justified as part of any health screening process.

Musculoskeletal symptom reporting was also associated with poorer performance on several questionnaires designed to assess quality of life, mood, executive function and memory. Only SF36 PCS, however, fell below the normative value of 50 (standard deviation (SD) 10) [10]. The population norm for SF36 MCS is also 50 (SD 10), and the values from this study are within this range. Values from normal populations are also available for the other measures used: HADSa: median 5, IQR 3 to 8; HADSd: median 3, IQR 1 to 5 [21]; PRMQ: mean 38.9, SD 9.2 [15]; CFQ: mean 43.5, SD 17.0 [22]; DEX: mean 20.8, SD 9.6 [23]. Although associated with musculoskeletal complaint in divers, at group level, the scores for these measures did not depart from the norm. Further, while an association between indicators of musculoskeletal dysfunction and physical quality of life is logically to be expected, an association with the other factors identified here is less so. Depression and anxiety have been associated with musculoskeletal pain in patients attending an outpatient rehabilitation clinic [12] using the HADS questionnaire. Unlike this study, however, scores were outside the normative range for both anxiety and depression. Pain has also been associated with disruption of cognitive function, attention and memory [24-26]. The level of pain considered in these studies, however, was greater than that in the present study, being sufficient to cause victims to seek medical treatment and associated with significant departures from normality. While there may be an association between pain and the factors studied, an alternative hypothesis might be considered more likely at the level of effect seen here. The divers that expressed symptoms of moderate to severe musculoskeletal symptoms were more likely to express symptoms of any kind. It might be expected, therefore, that any questionnaire which required them to report symptoms would return a higher score than in a control population. Rather than being a specific indicator of any dysfunction associated with pain, therefore, higher scoring might well be a non-specific response indicating more health-related concern and somatisation in this group. This hypothesis is supported by the absence of any association with more objective measures of memory and executive function by formal neuropsychological testing.

There were occupation-related risk factors for moderate to severe musculoskeletal symptoms identified in the study. Musculoskeletal symptoms were more likely in divers with experience in police and oilfield diving and in divers who had stopped diving. Police officers are exposed to a number of work-related stress factors [27] that might be expected to increase any tendency to somatise [28], and UK oilfield divers are known to have a greater tendency to express symptoms than other divers without any overt indication of any underlying illness [6]. Retiral from diving in this group of UK divers indicates a change in job status rather than a complete retiral from work, and retraining, a drop in income or a move to a less secure job may be factors underlying increased somatisation for this group.

Not only was general symptom reporting associated with musculoskeletal symptoms, it was also associated with a positive physical examination for divers. This effect was only seen, however, where the physical abnormality included pain or tenderness. This might be expected since pain identified on physical examination is yet another symptom which would be commoner in a group which had a tendency more readily to report symptoms of any kind. This concept is supported to some degree by the observation that the reduction in SF36 PCS associated with a positive physical examination was less significant in divers than in offshore workers, indicating that the abnormalities identified were not physically as important. The results of physical examination might be considered to offer an objective measure in the assessment of musculoskeletal symptoms. It is clear, however, that this may not be the case and that any subjective response from the persons examined needs to be considered alongside an assessment of their threshold for expressing symptoms of any kind. This may well be true for other modalities of physical examination. Neurological examination, in particular, requires a high degree of subjective response from the patient, and the effect identified here might underlie the high prevalence of unexplained minor physical findings on neurological examination in a sample of Norwegian divers [29]. This Norwegian study used observers who were not blinded to subject identity and medical history, and any tendency for the participant to influence the outcome of a physical examination would have been greater than in the present study.

Clearly, divers are more likely to experience symptoms than the control group, and positive findings on physical examination were related to this tendency. There was no indication, however, that symptom reporting necessarily indicated any significant medical problem. This study did not examine whether participants perceived that symptoms were related to any occupational factors, but in the UK, there is no strong perception that problem-free diving is injurious to health. The same is not true in Norway. Out of a population of 375 Norwegian divers working in the offshore oil and gas industry prior to 1990, at least 104 have been referred to a specialist hospital-based unit for the investigation of perceived diving-related problems [30]. Unsurprisingly, health-related quality of life scores were below population norms, but there was no indication of any association with observable current abnormality or disease. This population also reported a high prevalence of neurological and musculoskeletal symptoms and had a higher than expected number of people on disability benefit in a Government-sponsored commission report [5]. The release of this report was followed by the launching of a generous compensation scheme for oilfield divers with diving-related injury and medico-legal action against the Norwegian Government by a number of divers. It may be that some divers have a tendency to somatise and that when certain psychosocial pressures are applied, this converts to overt illness. Accordingly, UK divers with a tendency to somatise may be at the same risk, and any exercise, such as unnecessary screening, may further increase this risk by generating increased health-related anxiety.

This study may be considered to have weakness and strengths. The cross-sectional design does not allow any attribution of cause. A main purpose of the study, however, was to use physical examination to clarify the degree to which symptom reporting reflected physically identifiable disorder in the groups studied. Although the groups studied were randomly sampled from the background population, there may have been a degree of responder bias since we identified anxiety as a possible basis of symptomatic complaint and this is known to be associated with earlier presentation of diseases such as cancer [31]. From this, it might be expected that anxious people would be more likely to attend for examination and that they would, accordingly, be over-represented. Random sampling with a control group from an equivalent industry, however, allowed for such responder bias. Somatisation was not assessed using one of the standard questionnaires for this purpose, and this might be seen as a shortcoming. The standard instruments, however, would have been inappropriate for this study’s purpose since joint or limb pain symptoms are included in all the most commonly used questionnaires [20]. In fact, the scoring system used in the study covers all the symptoms generally elicited in somatisation questionnaires and allowed their assessment in the context of a standard medical examination, thus avoiding unnecessary repetition. The general symptom score used did not permit any putative diagnosis of somatoform disorder since it has not been validated. This was not one of the study aims, however, since our initial questionnaire study did not indicate a degree of symptom reporting that could be taken to indicate a somatoform disorder [6].

Medical examination does not provide a ‘gold standard’ for the identification of physical issues since abnormal physical signs are not necessarily associated with symptoms of pain or loss of function at the time it is conducted and, as detected here, it can be influenced by the subjective responses of the person examined. We wished, however, to study the basis of a range of musculoskeletal complaints compatible with a wide aetiology but predominantly manifesting as pain or discomfort, and the choice of a standardised, objective examination to provide this is justifiable [32]. A blinded physical examination technique minimised participant-doctor interaction as a source of bias in the demonstration of physical signs, and the use of doctors from outside the study team avoided the impact of any researcher preconceptions. In spite of these precautions, it was clear that participants with a tendency to express symptoms could influence the outcome of the examination.

In summary, questionnaire data reflected the prevalence of physically identifiable musculoskeletal abnormalities in divers and non-divers, but this relationship was not strong enough to offer a basis for future health screening. Musculoskeletal symptom reporting was also linked to a general tendency to express symptoms of any kind and to have less favourable scores on questionnaires for mood, memory and executive function. Scores, however, did not depart from the normal range, and there was no underlying abnormality of tests of neuropsychological function. It is suggested that high scoring on symptom questionnaires is an expression of a tendency to report symptoms of any kind or somatisation rather than any indication of abnormality in the modality addressed by the questionnaire. Multiple symptom reporting was also related to a positive physical examination when the abnormality detected included pain or tenderness. It is suggested that multiple symptom reporting may indicate a tendency to respond positively to subjective elements of a physical examination and that this effect may underlie the high prevalence of minor physical abnormalities detected in divers in other studies.

## Conclusions

Although musculoskeletal symptoms reflected the underlying prevalence of objectively identifiable disorders, the relationship was not strong enough for questionnaire data to be used for health surveillance of musculoskeletal disease. Musculoskeletal symptom reporting in divers was related to a general tendency to report symptoms of any kind, and this tendency may lead to a lower threshold for eliciting signs of pain or tenderness on physical examination. There is a risk for studies on divers, therefore, to make falsely high estimations of the level of musculoskeletal problems.

Claims for possible health effects of diving at work should consider the tendency in professional divers to express symptoms of any kind at a higher rate than in other working groups such as offshore workers. The importance of this study is that while it confirms previous reports of a higher rate of musculoskeletal symptom reporting in divers, it indicated that this higher rate need not be associated with abnormality or a greater prevalence of any physical disease.

## Abbreviations

95% CI: 95% confidence interval; CANTAB: Cambridge Neuropsychological Test Automated Battery; CFQ: Cognitive Failures Questionnaire; CVLT: California Verbal Learning Task; DEX: dysexecutive syndrome questionnaire; ExecComp: composite score reflecting executive function; GSS: general symptom score; HADSa: Hospital Anxiety and Depression Scale for anxiety; HADSd: Hospital Anxiety and Depression Scale for depression; LM: Logical Memory; MemComp: composite score reflecting memory performance; MSS: musculoskeletal symptom score; OR: odds ratio; PRMQ: Prospective and Retrospective Memory Questionnaire; SF36 MCS: Short Form 36 mental component score; SF36 PCS: Short Form 36 physical component score; SPSS: Statistical Package for the Social Sciences; WASI: Wechsler Abbreviated Scale of Intelligence.

## Competing interests

The authors declare that they have no competing interests.

## Authors’ contributions

JASR helped conceive of the study, designed and supervised the medical examination, participated in the design and coordination and drafted the manuscript. JIM helped conceive of the study and was the project manager. CLR helped conceive of the study and was responsible for the psychological questionnaire administration and assessment. SJJ helped conceive of the study and participated in its design and implementation. JRC participated in the design and quality assurance of psychological questionnaire administration and assessment. All the authors have read the paper and have provided criticism and feedback. All authors read and approved the final manuscript.
